# Autophagy inhibition reduces chemoresistance and tumorigenic potential of human ovarian cancer stem cells

**DOI:** 10.1038/cddis.2017.327

**Published:** 2017-07-20

**Authors:** Anna Pagotto, Giorgia Pilotto, Elena Laura Mazzoldi, Maria Ornella Nicoletto, Simona Frezzini, Anna Pastò, Alberto Amadori

**Affiliations:** 1 Department of Surgery, Oncology and Gastroenterology, University of Padova, Padova, Italy; 2 Istituto Oncologico Veneto-IRCCS, Padova, Italy

## Abstract

Epithelial ovarian cancer (EOC) is one of the most malignant gynecological tumors with a high mortality rate owing to tumor relapse after anticancer therapies. It is widely accepted that a rare tumor cell population, known as cancer stem cells (CSC), is responsible for tumor progression and relapse; intriguingly, these cells are able to survive nutrient starvation (such as *in vitro* culture in the absence of glucose) and chemotherapy treatment. Recent data also indicated that chemotherapy resistance is associated with autophagy activation. We thus decided to investigate both *in vitro* and *in vivo* the autophagic activity and the effects of the perturbation of this pathway in CSC isolated from EOC ascitic effusions. Ovarian CSC, identified according to their CD44/CD117 co-expression, presented a higher basal autophagy compared with the non-stem counterpart. Inhibition of this pathway, by *in vitro* chloroquine treatment or CRISPR/Cas9 ATG5 knockout, impaired canonical CSC properties, such as viability, the ability to form spheroidal structures *in vitro*, and *in vivo* tumorigenic potential. In addition, autophagy inhibition showed a synergistic effect with carboplatin administration on both *in vitro* CSC properties and *in vivo* tumorigenic activity. On the whole, these results indicate that the autophagy process has a key role in CSC maintenance; inhibition of this pathway in combination with other chemotherapeutic approaches could represent a novel effective strategy to overcome drug resistance and tumor recurrence.

Epithelial ovarian cancer (EOC) is the leading cause of death from gynecological malignancies and the fifth leading cause of all cancer-related deaths among women in the Western world.^1^ Early diagnosis of ovarian carcinoma has proved difficult to achieve, largely owing to lack of an identified pre-malignant precursor lesion, and owing to the anatomical location of the ovaries.^2^ Indeed, the symptoms associated with this malignancy are shared with several other more common gynecologic, gastrointestinal and urinary pathologies. To date, no validated screening test exists as CA-125 dosage, pelvic and transvaginal sonography have very low sensitivity and specificity.^3^ As a consequence, ~75% of patients present with signs of metastatic spread at the time of diagnosis, and ~80% of women with advanced disease have a 5-year survival rate of only 30%.^4^ In the last two decades, much effort has been spent in employing more effective surgery and combination treatment regimens, typically platinum- and taxane-based, resulting in complete response in 70% of patients.^5^ Despite these results, most patients relapse within 18 months with chemo-resistant disease.

One emerging model for the development of drug-resistant carcinomas suggests that a pool of self-renewing malignant progenitor cells exists. These rare cancer-initiating cells, also named cancer stem cells (CSC), present several features that confer chemoresistance, such as the expression of membrane efflux transporters, enhanced DNA repair and low mitotic index.^6^ Therefore, eradication of the stem cell compartment of a tumor might be the essential and most effective way of curing cancer and allowing long-lasting remission.

Recent studies have also revealed metabolic reprogramming as a new hallmark of cancer. In fact, mutations in cancer genes and alterations in metabolic signaling pathways frequently occur.^7^ Among these pathways, autophagy deregulation has been associated to tumor dormancy and resistance to treatment. Indeed, in the later stages of tumorigenesis an upregulation of autophagy may represent a mechanism of resistance to oxidative stress induced by chemotherapeutic drugs and may potentiate the survival to hypoxia and nutrient starvation^8^ resulting from the frequently defective tumor vascularization. Thus, we decided to evaluate the contribution of this pathway in CSC isolated from ascitic effusions of EOC-bearing patients. We previously demonstrated that ovarian CSC can be easily identified based on surface co-expression of CD117 (c-Kit) and CD44.^9^ These double-positive cells, compared with the CD44^+^CD117^−^ counterpart, are able to form spheroids, express stem cell-associated markers such as *NANOG*, *SOX2*, *OCT4*, as well as multidrug resistance pumps,^9^ and present higher tumorigenic potential when injected into immunocompromised mice, thus fulfilling the canonical requirements to be defined CSC.^10^

In the present study, we addressed the role of autophagy in the maintenance of the CD44^+^CD117^+^ cell pool; we found that autophagy is hyper-activated in ovarian CSC and that this aberrant activation may contribute to chemoresistance and tumor relapse. As a consequence, targeted therapies that specifically inhibit autophagy could represent an important resource to be used in combination with the conventional treatment of EOC.

## Results

### Ovarian CD44^+^CD117^+^ CSC display higher basal autophagy compared with bulk tumor cells

To investigate whether CSC could present different autophagy activation compared with tumor non-stem cells, we preliminarily confirmed that the co-expression of CD44 and CD117 is the most reliable marker for CSC identification in EOC. To this aim, we evaluated the mRNA expression levels of stemness-associated master genes *NANOG*, *SOX2* and *OCT4* in EOC cells FACS-isolated according to the expression of the most utilized markers in the literature: CD133,^11^ CD24,^12^ ALDH^13^ or CD44/CD117. Although CD24 was excluded from the analysis since it was expressed by most tumor cells in our ascitic effusion samples (Supplementary Figure S1A), CD44^+^CD117^+^ cells significantly overexpressed *NANOG*, *SOX2* and *OCT4*, compared with the negative counterpart CD44^+^CD117^−^ (Supplementary Figure S1D). No differences were detected in mRNA expression levels of these genes between CD113^+^ and CD133^−^ or ALDH^pos^ and ALDH^neg^ cells (Supplementary Figure S1B-C), thus supporting our choice of CD44/CD117 co-expression as CSC marker in EOC.

**Figure 1:**
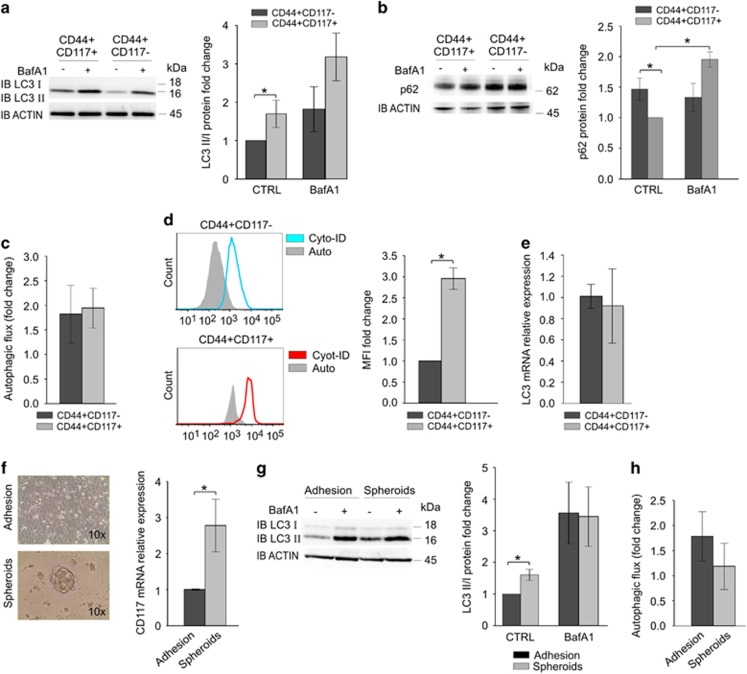
CD44^+^CD117^+^ ovarian CSC show higher basal autophagy than their CD44^+^CD117^−^ non-stem counterpart. (**a, b**) WB analysis of LC3-II/LC3-I ratio (**a**) or p62 (**b**) protein expression in FACS-sorted CD44^+^CD117^+^ and CD44^+^CD117^−^ cells from primary samples of EOC ascitic effusions. After sorting, cells were either treated with 100 nM BafA1 for 2 h or left untreated. Signal intensities of the LC3-II, LC3-I and p62 bands were quantified by scanning densitometry, and normalized against the actin signal. The graph on the right shows mean expression ratios±S.D. from four different experiments. *P<0.05. (**c**) The autophagic flux was calculated dividing LC3-II normalized signal intensity of BafA1-treated cells by the signal intensity of untreated cells for each cell subpopulation. The bar shows mean±S.D. from four different experiments. (**d**) Flow cytometry analysis of autophagic activity in CD44^+^CD117^+^ and CD44^+^CD117^–^ cells from EOC ascitic effusions and PDX. The cells were labeled with anti-CD44, anti-CD117 antibodies and Cyto-ID Autophagy detection kit. One representative experiment out of seven is shown (left panel). The graph shows the mean fluorescence intensity (MFI)±S.D. calculated from seven experiments (right panel). *P<0.05. Auto=unstained cells; Cyto-ID= stained cells. (**e**) qRT-PCR analysis of LC3 mRNA expression in FACS-sorted CD44^+^CD117^+^ and CD44^+^CD117^−^ cells. Shown are mean relative expression values in CD44^+^CD117^+^ cells compared with CD44^+^CD117^−^ cells (±S.D.) measured in four samples. (**f**) EOC primary and PDX cells were analysed by qRT-PCR for the expression of CD117. Shown are mean relative expression values (±S.D.) in samples cultured in spheroid-forming conditions compared with the same samples cultured in adherent conditions (*n*=4). *P<0.05. (**g**) WB analysis of LC3-II/LC3-I ratio protein expression in adherent cells *versus* spheroid cells from EOC effusions (left panel). Cells were either treated with 100 nM BafA1 for 2 h or left untreated. Signal intensities of the LC3-II and LC3-I bands were quantified by scanning densitometry and normalized against the actin signal. The graph shows mean expression ratios±S.D. from four consecutive experiments (right panel). (**h**) The autophagic flux was calculated by dividing LC3-II normalized signal intensity of BafA1-treated cells by the signal intensity of untreated cells for each cell subpopulation. The bar shows mean±S.D. from four experiments

Next, we evaluated the autophagic flux of ovarian CSC. Autophagy is invariably associated with the conversion of the microtubule-associated protein LC3 from its cytosolic form (LC3-I) to its autophagosome-associated form (LC3-II).^14^ Hence, we analyzed by western blotting (WB) the levels of LC3-II in FACS-sorted CD44^+^CD117^+^ and CD44^+^CD117^−^ cells from primary samples of ascitic effusions, collected from patients affected by EOC (Table 1). We took advantage of the autophagy inhibitor bafilomycin, which blocks the fusion of autophagosomes with lysosomes and therefore allows us to clamp autophagosome consumption, as previously described.^14^ As shown in Figure 1a, CD44^+^CD117^+^ cells presented a more active basal autophagy compared with CD44^+^CD117^−^ cells, as represented by the significantly higher *ex vivo* levels of LC3-II in basal conditions. Treatment with bafilomycin A1 (BafA1) induced in both cell populations an increase in LC3-II (Figure 1a). The different basal autophagy activation between CSC and non-CSC was confirmed by protein level analysis of p62, a well-known target of autophagy. Indeed, p62, also known as sequestosome 1, binds ubiquitinated protein aggregates within the autophagosomes, contributing to their lysosomal degradation. When autophagy is inhibited, p62 levels increase, making it a useful marker for the autophagic flux.^15^ Results indicated that CD44^+^CD117^+^ cells present significantly lower levels of p62 compared with non-CSC counterpart (Figure 1b), meaning higher p62 degradation within the autophagosomes. However, the autophagic flux (calculated as LC3-II ratio between BafA1-treated and untreated cells) did not show any significant difference in the two cell subsets (Figure 1c). Autophagic activity was also analyzed by intracellular autophagosome staining with Cyto-ID autophagy kit and quantified by flow cytometry. The obtained results confirmed a significantly higher basal autophagic activity in CD44^+^CD117^+^ cells, as indicated by a higher MFI of CSC than non-CSC once subtracted the correspondent unstained control, thus corroborating the WB data (Figure 1d). Real-Time PCR performed on *ex vivo* sorted CD44^+^CD117^−^ and CD44^+^CD117^+^ did not highlight any difference in *LC3* mRNA (Figure 1e), indicating that the higher protein levels of LC3-II (Figure 1a) were likely not owing to gene upregulation in CSC but rather an indicator of enhanced autophagic activity.

We next took advantage of a spheroid-formation assay as a model to further study the autophagic flux in ovarian CSC-enriched population. Cancer cells obtained from primary EOC samples and patient-derived xenografts (PDX), generated by orthotopic injection of EOC cells into immunodeficient animals,^16^ were cultured for 2 weeks under stem cell conditions (as detailed in Materials and Methods). PDX fully recapitulate the composition of the corresponding primary samples, as well as the CSC theory (Supplementary Figure S2). The enrichment in CSC was measured by evaluating the mRNA expression levels of CD117 in cells maintained in normal (adhesion) and in stem cell culture conditions (spheroids) (Figure 1f). Both cultures were treated with BafA1 as above, and LC3-II protein levels compared with the corresponding untreated samples. As demonstrated in FACS-sorted CSC, also the CSC-enriched spheroids presented higher basal autophagy compared with the adherent counterpart (Figure 1g). The autophagic flux, instead, calculated as the ratio between BafA1-treated and untreated cells, was again comparable in adherent cells and spheroids (Figure 1h).

Altogether, these experiments indicate that both *ex vivo*-derived and PDX-derived ovarian CSC show a prominent autophagic activity, compared with the bulk of tumor cells.

### Inhibition of autophagy affects canonical CSC properties

**Figure 2:**
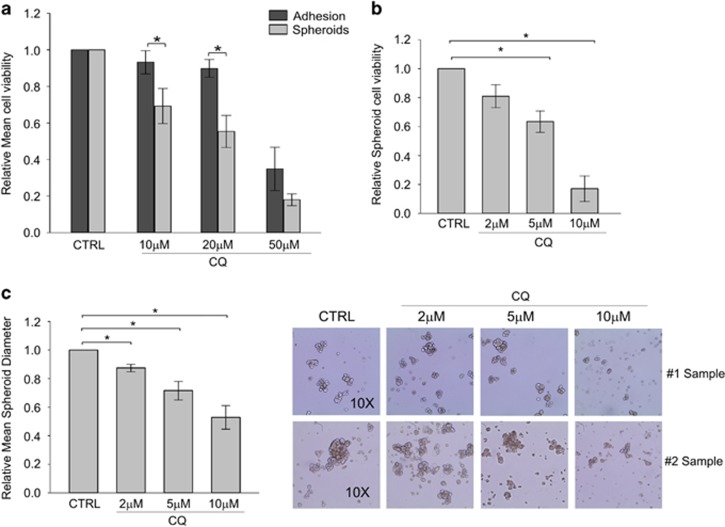
Autophagy inhibition by chloroquine reduces *in vitro* spheroid-forming ability of ovarian CSC. (**a**) Cell viability analysis by Live/Dead staining of EOC ascitic effusion cells cultured either in adherent or spheroid-forming conditions for 2 weeks and then treated with chloroquine (CQ, 10, 20 or 50 *μ*M) for 72 h. The graph represents the mean cell viability of three experiments normalized by the untreated cells (CTRL) for each culture condition. *P<0.05. (**b**) Cell viability analysis by Live/Dead staining of EOC effusion cells maintained for 1 week in spheroid-culture conditions in the presence of chloroquine (CQ, 2, 5 or 10 *μ*M). The bar shows mean±S.D. from three consecutive experiments. *P<0.05. (**c**) Spheroid diameter evaluation of EOC effusion cells cultured in the presence (CQ) or absence of chloroquine (CTRL). The bars on the left panel show mean±S.D. of treated cells normalized by the untreated ones of three different experiments. *P<0.05. On the right, representative pictures of spheroids obtained in the different culture conditions from two primary EOC samples

The interconnection between autophagy and maintenance of the CSC phenotype was further investigated by culturing EOC cells in CSC-enriched spheroid culture for 2 weeks. The cells were then treated with different concentrations of the autophagy inhibitor chloroquine (CQ; 10, 20 and 50 *μ*M) for 72 h. In parallel, chloroquine treatment was performed on the same samples cultured in adherent conditions. Interestingly, we observed a higher sensitivity to chloroquine treatment of spheroids (in terms of cell viability reduction) when compared with the counterpart maintained in adherent conditions (Figure 2a), suggesting that autophagy might be particularly important for the maintenance of ovarian CSC viability. In another set of experiments, in which chloroquine (2, 5 and 10 *μ*M) was added at the beginning of culture in stemness conditions and spheroid generation was evaluated 1 week later, we observed a dose-dependent cell viability reduction (Figure 2b) as well as a decrease in the mean diameter of the obtained spheroids (Figure 2c).

To further demonstrate the importance of autophagy in CSC maintenance, we took advantage of the CRISPR/Cas9 genome editing technique to stably knockout the autophagy-related gene-5 (ATG5) in an EOC cell line (OVCAR-3). ATG5 acts as an E3-ubiquitin ligase involved in the elongation of autophagosome, the initial step of the autophagic process. Cells lacking ATG5 fail to induce autophagy, resulting in impaired LC3-I conversion into LC3-II and accumulation of p62.

**Figure 3:**
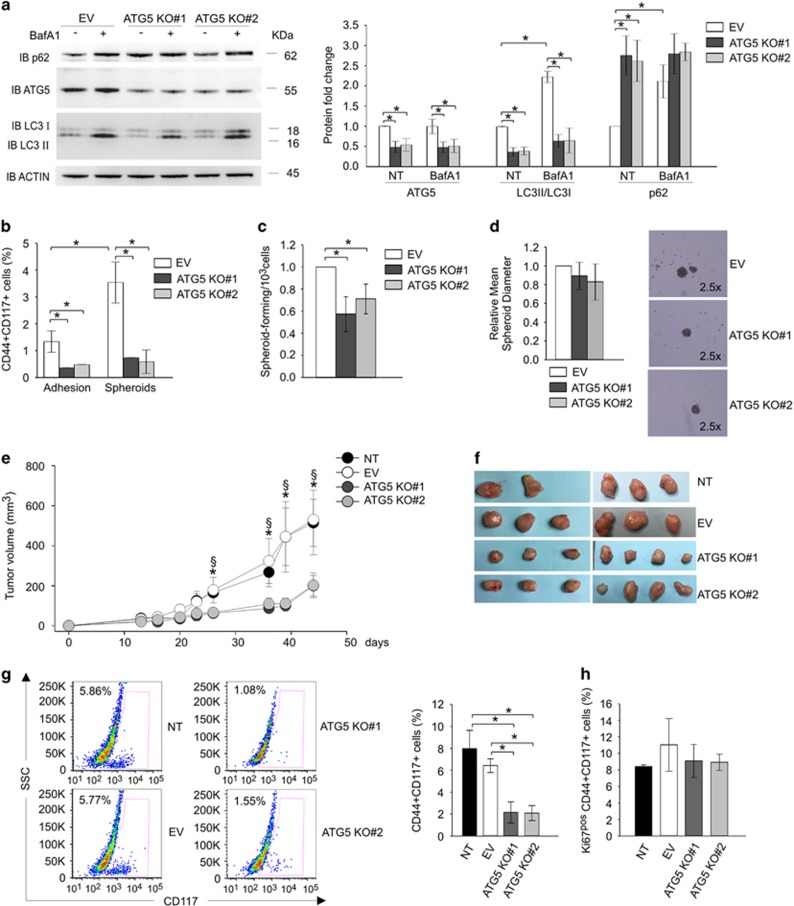
ATG5 is critical for CSC *in vitro* spheroid-forming ability and in *vivo* tumorigenic potential. (**a**) Western blot analysis of ATG5, p62 and LC3-II/LC3-I expression in CRISPR/Cas9 knockout OVCAR-3 cells either treated with 100 nM BafA1 for 2 h or left untreated. The cells were transduced with empty vector (EV), or two different constructs for ATG5 knockout (KO#1 and KO#2). Signal intensities of the bands were normalized against the actin signal. One representative blot is shown on the left panel; on the right panel, the graph shows mean expression ratios±S.D. from five different experiments. *P<0.05. (**b**) Flow cytometry analysis of CD44/CD117 co-expression in OVCAR-3 cells transduced with empty vector (EV), or two different constructs for ATG5 knockout (KO#1 and KO#2) after 2 weeks in normal (Adhesion) or stemness-culture conditions (Spheroids). Data are expressed as mean±S.D. from three experiments. *P<0.05. (**c**) Extreme Limiting Dilution Assay (ELDA) of EV, ATG5 KO#1 and ATG5 KO#2 OVCAR-3 cells. Data are expressed as mean±S.D. from three experiments. *P<0.05. (**d**) Spheroid diameter evaluation of EV, ATG5 KO#1 and ATG5 KO#2 OVCAR-3 cells. The mean spheroid diameter (±S.D.) from three experiments normalized by the EV is plotted in the left graph; representative pictures of spheroids are shown on the right. *P<0.05. (**e**) Tumor growth curves of non-transduced (NT), EV, ATG5 KO#1 and ATG5 KO#2 OVCAR-3 cells s.c. injected into NSG mice. Data are mean values±S.D.; *n*=5 for NT, *n*=6 for EV, *n*=7 for ATG5 KO#1 and #2; *P<0.05. *ATG5 KO#1, ATG5 KO#2 *versus* NT; ^§^ATG5 KO#1, ATG5 KO#2 *versus* EV. (**f**) Representative pictures of tumors at the end of the experiments (45 days). (**g**) *Ex vivo* flow cytometry analysis of CD117 expression within the CD44^pos^ cells isolated from tumors generated from non-transduced (NT), EV, ATG5 KO#1 and ATG5 KO#2 OVCAR-3 cells s.c. injected into NSG mice. On the left representative plots; on the right the histogram showing the mean values±S.D.; *n*=5 for NT, *n*=6 for EV, *n*=7 for ATG5 KO#1 and #2; *P<0.05. (**h**) Histogram showing the mean values±S.D. of e*x vivo* flow cytometry analysis of Ki67 expression within CD44^+^CD117^+^ cells isolated from NT, EV, ATG5 KO#1 and #2 tumors. *n*=5 for NT, *n*=6 for EV, *n*=7 for ATG5 KO#1 and #2

In our setting, WB analysis demonstrated that the reduction of ATG5 protein levels in OVCAR-3 cells was obtained only with two constructs (ATG5 KO#1 and KO#2) out of three tested (Supplementary Figure S3A). The ATG5-knockout cells presented significantly lower LC3-II protein levels and a correlated accumulation of p62 compared with cells transduced with the empty vector (EV; Figure 3a). Accordingly, treatment with BafA1 increased LC3-II/LC3-I ratio and p62 protein levels in control OVCAR-3 cells, whereas only a slight but not significant increase of the ratio was detected in ATG5 KO#1- and #2-transduced cells after 2 h of BafA1 treatment (Figure 3a).

We then evaluated the effects of ATG5 silencing on CSC ability to form spheroidal structures *in vitro*. After 2 weeks in stemness-culture conditions we observed an expansion of the CSC compartment in OVCAR-3 cells transduced with the EV (Figure 3b) compared with cells maintained in adhesion. On the contrary, the percentage of CD44^+^CD117^+^ cells only slightly increased in ATG5 KO#1- and ATG5 KO#2-transduced cells under spheroid-forming culture condition. In both culture conditions, the percentage of double-positive cells in ATG5-knockout cells was significantly lower compared with EV cells (Figure 3b). In line with this finding, Extreme Limiting Dilution Assay (ELDA) analysis demonstrated that autophagy inhibition by stable gene silencing was correlated with a reduction in the ratio of spheroid-forming cells (Figure 3c). However, the ATG5 knockout was not associated with an alteration in the mean spheroid diameter (Figure 3d), indicating that autophagy blockade may interfere with CSC self-renewal rather than proliferation. Indeed, cell cycle analysis and colony formation assay performed in ATG5-knockout cells maintained in adhesion culture conditions did not show any difference between EV, ATG5 KO#1- or KO#2-transduced OVCAR-3 cells (Supplementary Figure S3B, S3C and S3D). Finally, we evaluated the effects of ATG5 silencing on CSC tumorigenic potential. To this end, we injected subcutaneously (s.c.) non-transduced (NT), EV- or ATG5 KO#1- and KO#2-transduced OVCAR-3 cells into immunodeficient mice. As shown in Figures 3e and f, tumors from ATG5 knockout cells grew more slowly than those obtained from NT cells or cells transduced with the EV. *Ex vivo* flow cytometry analysis revealed a significant reduction of the CD44^+^CD117^+^ cell percentage in ATG5-KO tumors compared with NT and EV-transduced ones (Figure 3g). However, no difference was detected in the proliferative potential of CSC, as demonstrated by the evaluation of Ki67 expression (Figure 3h).

Altogether, these results show that autophagy blockade by different pharmacologic and genetic approaches severely impairs canonical CSC properties, such as the ability to form spheroids and the efficiency of tumor generation in immunodeficient animals, without affecting CSC proliferation.

### Autophagy blockade reduces CSC ability to resist *in vitro* and *in vivo* chemotherapy treatment

**Figure 4:**
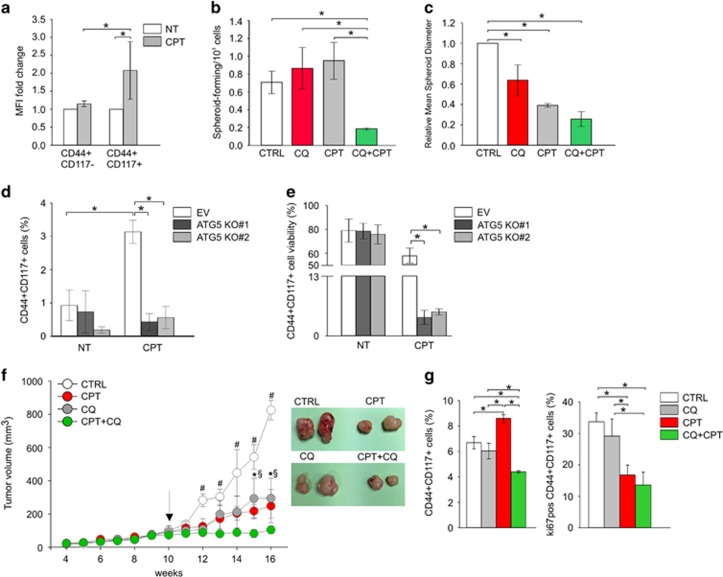
Autophagy blockade impairs CSC resistance to chemotherapy treatment. (**a**) Flow cytometry analysis of autophagic activity in CD44^+^CD117^+^ and CD44^+^CD117^−^ cells by Cyto-ID Autophagy detection kit. Cells were either treated *in vitro* with carboplatin (20 *μ*g/ml) for 72 h or left untreated. Data are expressed as Mean Fluorescent Intensity (MFI)±S.D. calculated from three experiments *P<0.05. (**b**) Extreme Limiting Dilution Assay (ELDA) of EOC ascitic effusion cells, isolated from both primary and PDX samples, following different treatment regimens. FACS-sorted alive tumor cells were cultured *in vitro* for 72 h under normal culture conditions in the presence of CQ (20 *μ*M), CPT (20 *μ*g/ml) or the combination of the two drugs, and subsequently plated in spheroid-forming conditions for ELDA. Data are expressed as mean±S.D. from three consecutive experiments, *P<0.05. (**c**) Spheroid diameter evaluation of EOC effusion cells, isolated from both primary and PDX samples, following different treatment regimens, as described in **c**. The mean spheroid diameter (±S.D.) from three experiments normalized by the untreated cells (CTRL) is plotted in the graph. *P<0.05. (**d**–e) Flow cytometry analysis of CD44/CD117 co-expression (**d**) and cell viability (**e**) in OVCAR-3 cells transduced with empty vector (EV), or two different constructs for ATG5 knockout (KO#1 and KO#2), treated *in vitro* for 72 h with carboplatin (20 *μ*g/ml) or left untreated. Data are expressed as mean±S.D. from three experiments. *P<0.05. (**f**) Tumor growth curves in NSG mice treated with saline solution (as a control, CTRL), carboplatin (CPT, 50 mg/Kg weekly), chloroquine (CQ, 100 mg/Kg every 2 days) or the combination of the two, after s.c. injection of EOC effusion cells from PDX samples. Data are mean values±S.D. of six tumors/group. *P*<0.05.*CPT+CQ *versus* CQ alone; ^§^CPT+CQ *versus* CPT alone; ^#^CPT+CQ *versus* CTRL. The arrow indicates when treatment started. On the right representative pictures of tumors (two tumors/group) at the end of one (out of three) experiments performed. (**g**) *Ex vivo* flow cytometry analysis of CD44/CD117 co-expression (left panel) and Ki67 (right panel) in tumor harvested from mice treated with saline solution (CTRL), carboplatin (CPT), chloroquine (CQ) or the combination of the two drugs (CPT+ CQ). Data are expressed as mean±S.D. from four tumors/group. *P<0.05

Another feature of CSC, which represents an important limit to conventional therapy, is their ability to resist chemotherapeutic treatment. It has been reported that autophagy is induced in established ovarian cancer cell lines in response to platinum salts as a survival mechanism, resulting in a sensitization of the cells to the treatment when autophagy is suppressed.^17^ Thus, we evaluated carboplatin-mediated autophagy activation by intracellular autophagosome staining in primary EOC and PDX samples. As shown in Figure 4a, 72 h of *in vitro* carboplatin treatment induced a significant MFI increase in CD44^+^CD117^+^ cells.

Therefore, we addressed the role of autophagy in stress conditions by evaluating the effect of carboplatin, alone and in combination with chloroquine, on the spheroid-forming ability of tumor cells from EOC patients. To this end, EOC cells derived from primary samples or PDX were pulsed for 72 h with carboplatin, chloroquine or the combination of the two; subsequently, equal numbers of live cells were plated in spheroid-forming conditions according to the ELDA protocol (see Materials and Methods). As shown in Figure 4b, pre-treatment of tumor cells with the autophagy inhibitor chloroquine or with carboplatin alone did not cause any significant change in the spheroid-forming ratio, compared-to-untreated cells. On the contrary, pre-treatment for 72 h with a combination of carboplatin and chloroquine caused a dramatic decrease in the number of spheroid-forming cells (Figure 4b). In addition, the mean spheroid diameter was lower in samples pre-treated with chloroquine or carboplatin alone or the combination of the two (Figure 4c). These data indicate that autophagy is a key mechanism exploited by ovarian CSC to survive carboplatin treatment. To better demonstrate the efficacy of carboplatin-chloroquine combined treatment, we determined the combination index (CI) in primary and PDX samples (as described in Materials and Methods). As reported in Supplementary Table S1, the results indicated a synergistic effect of the two drugs when used at the concentration of 20 *μ*g/ml (for carboplatin) and 20 *μ*M (for chloroquine). Similar conclusions could be drawn from experiments in which ATG5 knockout OVCAR-3 cells were treated *in vitro* with carboplatin (20 *μ*g/ml). Treatment of control cells transduced with the EV induced, as previously reported, a statistically significant selection of double-positive, drug-resistant CSC (Figure 4d). On the contrary, carboplatin treatment was not associated with an increase in the percentage of CD44^+^CD117^+^ cells in ATG5 KO#1- and ATG5 KO#2-transduced cells, thus indicating that autophagy blockade increases CSC platinum sensitivity (Figure 4d). Indeed, cell viability of ATG5 KO#1 and #2 CD44^+^CD117^+^ cells was significant reduced by carboplatin treatment, compared with CSC from EV-transduced cells (Figure 4e).

Finally, to evaluate the effect of autophagy inhibition on the CSC tumorigenic potential, PDX cells were injected s.c. into NSG mice. The animals were then treated with different therapeutic regimens: saline solution (as a control), chloroquine, carboplatin or a combination of the two. Both treatments with carboplatin and chloroquine alone significantly slowed tumor growth, compared with control. Strikingly, combination treatment had a synergistic effect inducing an even more pronounced tumor growth reduction (Figure 4f). Again, *ex vivo* analysis demonstrated that autophagy can represent a survival mechanism adopted by CSC to resist chemotherapy treatment. Indeed, the percentage of CD44^+^CD117^+^ cells was significantly lower in tumor harvested from combined-treated mice (carboplatin+chloroquine) compared with untreated or single-treated mice (Figure 4g, left panel). Moreover, *ex vivo* Ki67 analysis within the CD44^+^CD117^+^ compartment further demonstrated that autophagy blockade did not affect CSC proliferation (Figure 4g, right panel).

## Discussion

One of the major hurdles in EOC treatment is to overcome tumor relapse. By now, it is largely accepted that tumor growth, progression and relapse are sustained by CSC,^18^ a small cell subset able to resist both chemo- and radio-therapy treatment *in vitro* and *in vivo*. Several mechanisms may be involved in CSC resistance, including the ability to enter quiescence, and the expression of multidrug resistance pumps or detoxifying enzymes; in the last decade, the activation of autophagy has also been identified as a survival mechanism potentially exploited by CSC. The term ‘autophagy’ defines a cellular process of lysosomal degradation of self-components. It refers to a multistep mechanism that has been thoroughly characterized at the molecular level (reviewed by Yang and Klionsky^19^). In cancer, autophagy plays a complex role depending on tumor stage, type and genetic background.^20^ In the early stages of tumorigenesis, it has a protective effect by preventing the accumulation of defective organelles, such as pro-oxidative mitochondria, which could enhance the rate of DNA mutation. Consistent with this, inactivating mutations of genes that positively regulate autophagy facilitate tumor formation. In later stages of tumor progression, autophagy activation seems to be a survival mechanism that counteracts the damage induced by chemotherapy or nutrient/oxygen starvation and sustains tumor growth by recycling of degraded metabolites.^21^ High autophagy levels have been detected in CSC derived from different solid and hematologic malignancies.^22, 23, 24, 25, 26^ However, the precise role of autophagy in cancer progression and the contribution of this pathway to CSC resistance to treatment is still unknown in ovarian tissue. Thus, we decided to evaluate autophagy activation and the effects of its perturbation in CD44^+^CD117^+^ CSC isolated from ascitic effusions collected from EOC-bearing patients.^9, 27, 28, 29^ *Ex vivo* analysis indicated that FACS-sorted CD44^+^CD117^+^ cells present a higher basal autophagy activation compared with the non-stem counterpart, as demonstrated by higher LC3-II protein levels and autophagosome staining, and lower p62 levels. Similarly, cells maintained in spheroid cultures, enriched in CSC, showed higher protein levels of the LC3-II marker of autophagosome formation (Figure 1).

The relevance of autophagy activation for the maintenance of canonical CSC features was first addressed by treating for 72 h EOC cell cultures with chloroquine, a known inhibitor of this pathway. Our results indicated that chloroquine affects CSC viability in a dose-dependent manner, inducing a stronger cell viability reduction in EOC cells cultured *in vitro* in spheroid-forming conditions compared with cells maintained in a differentiated/adhesion state. Moreover, treatment with lower doses of chloroquine for 1 week inhibited *in vitro* spheroid growth, as demonstrated by the reduction of spheroid diameter (Figure 2).

Next to chloroquine treatment, we knocked-out ATG5, involved in the regulation of the initial phase of the autophagy process. ATG5 knockout also impaired all the canonical features of CSC: indeed, ATG5-KO cells presented a lower percentage of CD44/CD117 co-expressing CSC, lower spheroid-forming ability and reduced tumorigenicity when injected s.c. into immunocompromised mice (Figure 3). Moreover, ATG5 silencing increased *in vitro* CSC sensitivity to chemotherapy as demonstrated by the significant reduction in the co-expression of CD44 and CD117 and their cell viability upon 72 h of carboplatin treatment (Figure 4).

However, unlike *in vitro* chloroquine treatment, the absence of ATG5 did not affect CSC proliferation, as demonstrated by cell cycle analysis, Ki67 expression, spheroid diameter evaluation and colony formation assay (Figure 3 and Supplementary Figure S3). These data indicated that autophagy is mainly involved in self-renewal/maintenance rather than in growth inhibition of CSC. The different results obtained with chloroquine treatment and ATG5 knockout could be in part explained by the autophagy-independent cytotoxic effect of chloroquine which has been reported to induce apoptosis both through p53-dependent and p53-indipendent mechanisms in melanoma and glioma cells.^30, 31^

As a whole, our results suggest that autophagy is profoundly involved into CSC regulation. Thus, to better demonstrate that autophagy inhibition could be a strategy to reduce CSC survival during anticancer treatment, we treated immunodeficient mice, s.c. injected with PDX cells, with carboplatin and chloroquine. Interestingly, the combination of the two treatments acted synergistically by significantly reducing tumor growth, compared with chloroquine or carboplatin alone (Figure 4). *Ex vivo* analysis demonstrated that such tumor growth delay was due to a reduction in the CSC compartment, rather than a reduction in CSC proliferative potential. The enhancement of chemotherapy effectiveness associated with chloroquine treatment has been already demonstrated in other tissues such as liver, pancreas, breast and colon,^32, 33, 34, 35, 36^ and our results suggest a possible clinical application of the combined therapy in the treatment of EOC: at the same time, this approach would offer the possibility to counteract tumor growth and impair the CSC compartment, thus reducing tumor relapse.

In conclusion, these results point to the combination of autophagy inhibition with anticancer treatment as a possible strategy to overcome the limits of current therapies in the eradication of EOC CSC population.

## Materials and methods

### Primary samples, cell lines and *in vitro* culture

This study was approved by the Institutional Ethics Committee for patient studies, according to the principles of the Declaration of Helsinki. Ascitic effusions were obtained from 40 EOC patients that provided written informed consent. Patients' clinical and pathologic features are summarized in Table 1. Tumor cells were isolated and maintained in RPMI-1640 medium supplemented with 10% FBS (GIBCO Invitrogen, Monza, Italy), 1% Penicillin/Streptomycin (Lonza, Basel, Switzerland), 1% sodium pyruvate (Lonza), and 1% l-glutamine (GIBCO). Cells were cultured at 37 °C, 5% CO_2_, and harvested at confluence using trypsin-EDTA (Invitrogen). OVCAR-3 cells were purchased from ATCC (Manassas, VA, USA) and cultured in the same conditions as primary samples. The cells were harvested, when 80–90% confluent, using Trypsin-EDTA (Invitrogen) and used within six months from resuscitation.

For spheroid-culture conditions, cells were plated in poly-2-hydroxyethyl methacrylate (PhEMA)-coated plates (Corning, NY, USA) in serum-free DMEM/F12 medium (Invitrogen) supplemented with bFGF (20 ng/ml, Peprotech, Rocky Hill, NJ, USA), EGF (20 ng/ml, Peprotech) and B27 (GIBCO) at a density of 5 × 10^4^ cells/well. Medium was replaced every 7 days. To evaluate spheroid-forming capacity and diameter modulation induced by chloroquine or carboplatin, cells were pre-treated in normal culture condition to avoid the effect of serum deprivation culture. After 72 h, cells were transferred to phEMA-coated plates as described above. Before evaluating LC3 protein levels and the cytotoxic effect of chloroquine on spheroid compared with adherent cells, the spheroid culture were kept in complete medium over-night.

For autophagy flux evaluation, BafA1 (Sigma-Aldrich, St Louis, MO, USA) was added to the cells for 2 h at the final concentration of 100 nM.^37^

According to experimental designs, cells were treated *in vitro* with chloroquine (CQ; Sigma-Aldrich) at the final concentration of 2, 5, 10, 20 or 50 *μ*M, or with carboplatin (CPT, 20 *μ*g/ml). For CI evaluation, cells were *in vitro* treated with different doses of chloroquine and carboplatin and their combination; after 72 h cell viability was evaluated by AnnexinV/PI staining and data were subjected to automatic calculation of CI using CompuSyn software.^38^

### Flow cytometry

The cells were stained with Live-Dead to discriminate living cells. The following anti-human antibodies were used: anti-CD44 (1:1,000; Abcam, Cambridge, UK), anti-CD117 (non-activating AC126 clone, 1:10; Miltenyi-Biotec, Bergish Gladbach, Germany), anti-CD45 (1:10; Miltenyi-Biotec) and anti-Ki67 (1:10; BD Bioscience, Franklin Lakes, NJ, USA). All the cytofluorimetric analyses were performed using a FACS LSRII (BD); data were collected from at least 1 × 10^5^ cells/sample and elaborated with FlowJo software (TreeStar, Ashland, OR, USA). For FACS sorting, antibody-labeled cells were separated with a MoFloAstrios Cell Sorter (Beckman Coulter, Brea, CA, USA); the purity of the sorted populations always exceeded 90%. To evaluate autophagic activity, EOC effusion cells were labeled with Cyto-ID Autophagy detection kit (Enzo Life Sciences).

For cell cycle analysis, cells were fixed with cold ethanol and then incubated for 1 h in a Dapi/RNAse solution. For cell viability analysis, cells were incubated for 15 min at 37 °C with AnnexinV/PI staining kit (Roche, Basel, Switzerland).

### WB

FACS-sorted CD44^+^CD117^+^ and CD44^+^CD117^−^ cells, cells cultured in adhesion or spheroid-forming conditions, or OVCAR-3 cells were lysed and subjected to SDS-PAGE and WB. Immunoreactivity was evaluated using the following antibodies: anti-actin (1:500; Sigma-Aldrich), anti-LC3B (1:1000; Cell Signaling Technology, Boston, MD, USA), anti-ATG5 (1:1000; Cell Signaling Technology) and anti-p62 (1:1000; Genetex, San Antonio, TX). The blots were hybridized with a 1:5000 dilution of HRP-conjugated anti-mouse or anti-rabbit antibody (Amersham-Pharmacia, Little Chalfont, UK), as appropriate. Finally, the signal was detected by chemiluminescence with SuperSignal kit (Pierce, Rockford, IL, USA), and lane densitometry analyzed by standard procedures.

### ELDA

To determine the frequency of spheroid-forming precursors, we performed an ELDA in EOC or OVCAR-3 cells. Cells were pre-treated in adhesion culture condition with chloroquine (20 *μ*M), carboplatin (20 *μ*g/ml), the combination of the two, or left untreated. After 72 h, the cells were counted and plated at different concentrations in 96-well flat-bottom ultra-low attachment PhEMA-coated plates (Corning) in a total volume of 0.1 ml of serum-free DMEM/F12 medium supplemented with B27 (Gibco), EGF (20 ng/ml, Peprotech) and bFGF (20 ng/ml, Peprotech). Thirty replicate wells were set up for each cell concentration. After 10 days of incubation, the wells were scored for spheroid formation; the frequency of spheroid-forming precursors in each population was calculated by ELDA web tool (http://bioinf.wehi.edu.au/software/elda). Data are expressed as the number of spheroid-forming cells/10^3^ cells.

### Colony formation assay

For colony formation assay, cells were plated at different densities under normal culture conditions in complete RPMI medium. After 10 days, cells were washed in PBS and then fixed with cold methanol for 10 min at +4 °C. Staining was performed with crystal violet 33% solution for 2 min at RT. Pictures were acquired with Leica EC3 microscope.

### RNA extraction, reverse transcription and quantitative PCR

Total RNA was extracted by the TRIzol method according to manufacturer’s instructions. The PCR step was performed using an ABI PRISM 7900HT Sequence Detection System (Applied Biosystems). Results were analyzed using the comparative ΔΔCt method; ΔΔCt values were utilized to calculate the RQ=2^−ΔΔCt^. Data were expressed as the fold difference in gene expression (normalized to the housekeeping gene *β*_2_-microglobulin) relative to a reference sample, as indicated in the individual figure legends. qRT-PCR efficiency ranged from 95% to 105%. Primer sequences were CD117: 5′-GGATTCCCAGAGCCCACAAT-3′ and 5′-GGCAGTACAGAAGCAGAGC-3′ B_2_-micro: 5′-TCTCTCTTTCTGGCCTGGAG-3′ 5′-TCTCTGCTGGATGACGTGAG-3′ LC3: 5′-AGACCTTCAAGCAGCGCCG-3′ and 5′-ACACTGACAATTTCATCCCG-3′

### CRISPR/Cas9 ATG5 knockout

Single Guide RNAs (sgRNAs) to ATG5 were designed using the online guide http://www.e-crisp.org/E-CRISP/designcrispr.html. The following sgRNAs were used: ATG5 KO#1 GTGCTTCGAGATGTGTGGTT, KO#2 GATCACAAGCAACTCTGGAT and KO#3 GGCCATCAATCGGAAACTCA, targeting, respectively, exon 2, 5 and 6. The LentiCRISPR v2 vector, gift from Feng Zhang (Addgene plasmid # 52961), was digested with BsmB1 and ligated with annealed sgRNAs.^39^

### *In vivo* studies

Severe combined immunodeficiency (NOD/SCID) and NSG mice were obtained from internal breeding. Procedures involving animals and their care were performed according to institutional guidelines that comply with national and international laws and policies (EEC Council Directive 86/609, OJ L358, 12 December 1987). PDX were generated by injecting intraperitoneally into NOD/SCID mice 5 × 10^5^ tumor cells from ascitic effusions of EOC patients.^16^

For chloroquine and carboplatin treatment, 5 × 10^5^ CD45^-^CD44^+^ tumor cells or OVCAR-3 cells were isolated by FACS sorting from high-grade serous ovarian cancer PDX, and injected s.c. in 200 *μ*l of Matrigel in both dorsolateral flanks of NSG mice. When tumors reached 100 mm^3^ volume, mice were randomized in four groups, and treated with chloroquine (100 mg/kg every 2 days), carboplatin (50 mg/Kg weekly), both drugs, or with equal saline amounts as a control. Tumor growth was evaluated by caliper measurements. Mice were killed when the tumors of the control group reached 600–800 mm^3^ volume.

### Statistical analysis

Data from replicate experiments were shown as mean values±S.D. Comparisons between groups were done by the two-tail Student’s *t*-test and Mann–Whitney test, as appropriate.

## Figures and Tables

**Table 1:** Clinical characteristics of EOC-bearing patients and association with the percentage of CSC

	***N* (% of total)**	**%CSC (range)**	***P*-value**^a^
*Histotype*			NS
Serous	36 (90)	2.05±2.0 (0.62–12.6)	
Mucinous	1 (2.5)	1.18 (–)	
Undifferentiated/clear cells	3 (7.5)	2.5±1.01 (1.5–3.50)	
			
*Stage*			NS
3A	1 (2.5)	2.69 (–)	
3B	4 (10)	1.29±1.6 (1.28–4.24)	
3C	24 (60)	1.59±0.5 (0.62–2.50)	
4	11 (27.5)	2.8±3.3 (0.8–12.6)	
			
*Grading*			NS
G1	3 (7.5)	2.05±1.2 (1.44–3.53)	
G2	0 (0)	–	
G3	37 (92.5)	2.09±1.95 (0.6–12.6)	
Total	40 (100)		

Abbreviation: NS, not significant

a *χ*^2^ test

## Supplementary Material

SupplementaryInformation

SupplementaryInformation
