# Supplementary material for: Autophagy inhibition reduces chemoresistance and tumorigenic potential of human ovarian cancer stem cells
Source: Cell Death Dis. 2017 Jul 20;8(7):e2943–. doi: 10.1038/cddis.2017.327 (PMC5550872; doi:10.1038/cddis.2017.327)
Supplement: SupplementaryInformation [file cddis2017327x1.doc]

**Supplementary Figures**

**
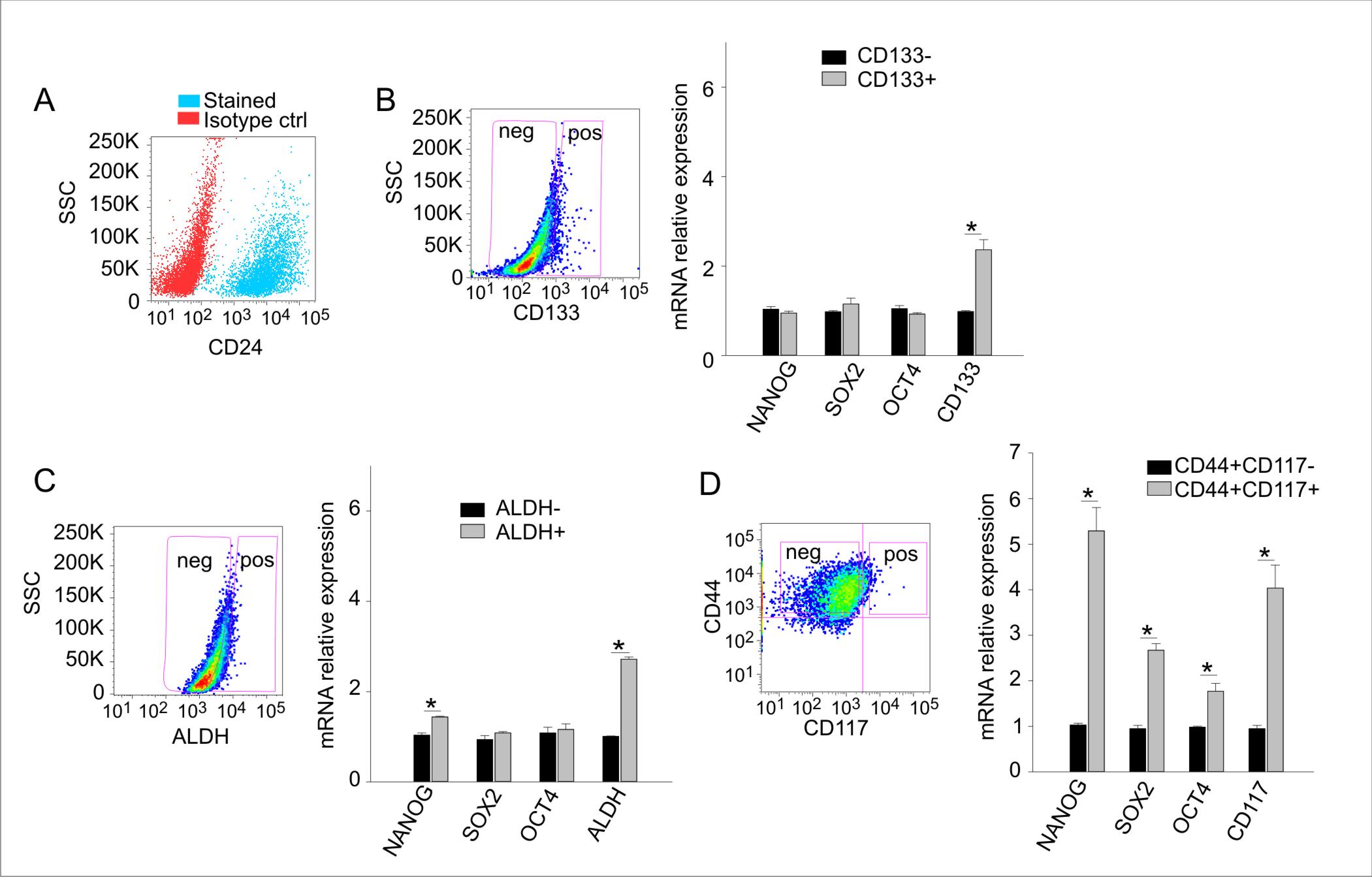
**

**Supplementary Figure S1. CD44/CD117 co-expression is the most reliable marker for EOC CSC.**

**A)** Flow cytometry analysis of CD24 expression in a representative EOC sample.

**B-D)** Representative flow cytometry analysis of CD133 expression (B), ALDH activity (C) and CD44/CD117 co-expression (D) in EOC samples on the left. In each panel, the right histogram shows mRNA levels of stemness-associated genes in EOC cells isolated by FACS according to the expression of the indicated marker. Data are shown as mean ± sd of 3 different samples; **P*<0.05

**
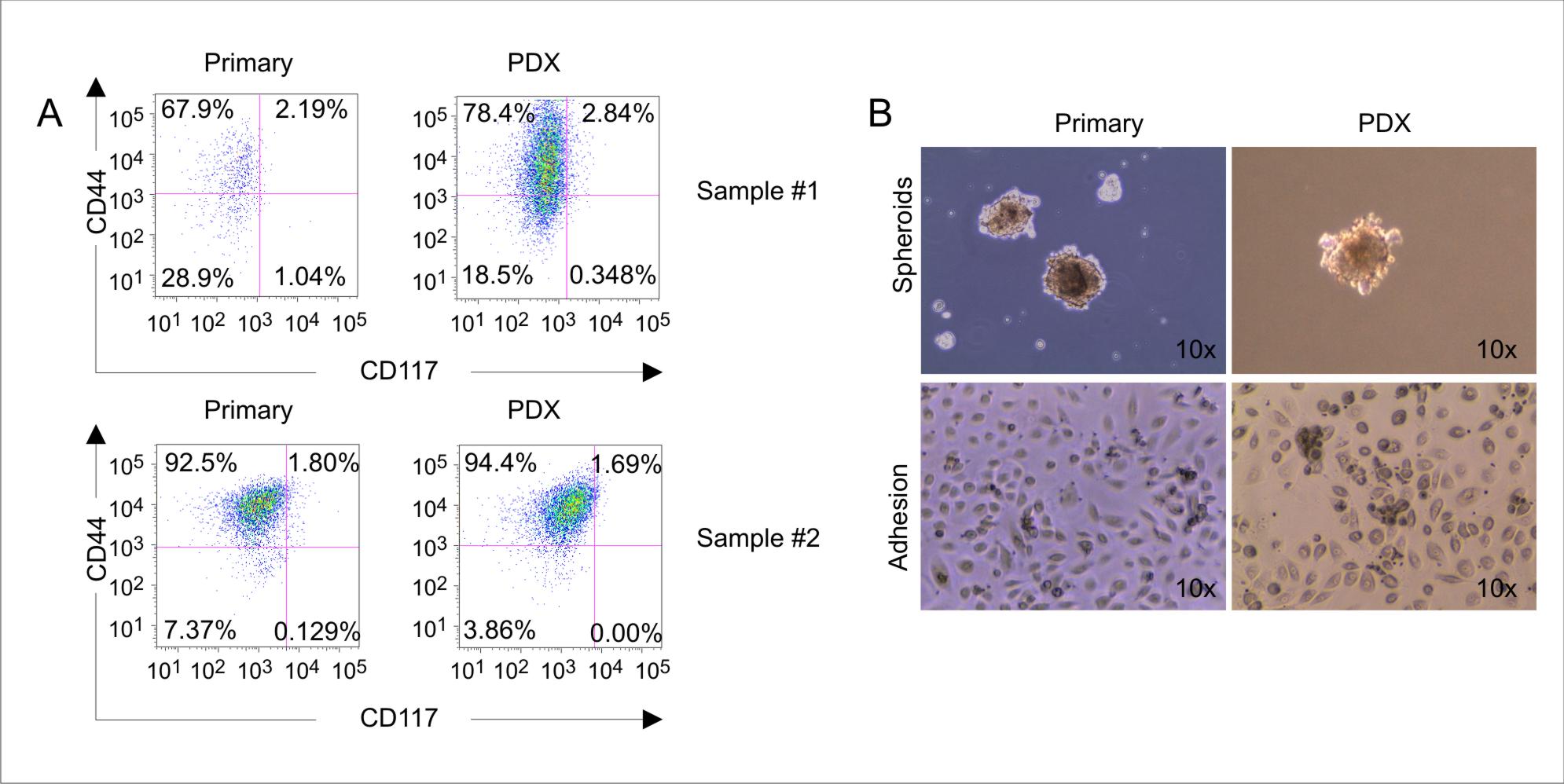
**

**Supplementary Figure S2. Patient-derived xenografts (PDX) maintained the CSC content and characteristics of primary samples.**

**A)** Flow cytometry analysis of CD44 and CD117 expression in two representative primary EOC samples (Primary) and their *primo vivo* xenograft (PDX) obtained by intraperitoneal injection of 0.5x10^6^ cells into immunocompromised NOD/SCID mice.

**B)** Representative picture of cells isolated from a primary sample and the corresponding PDX, maintained in Adhesion or pHEMA (Spheroids) culture conditions for 14 days.


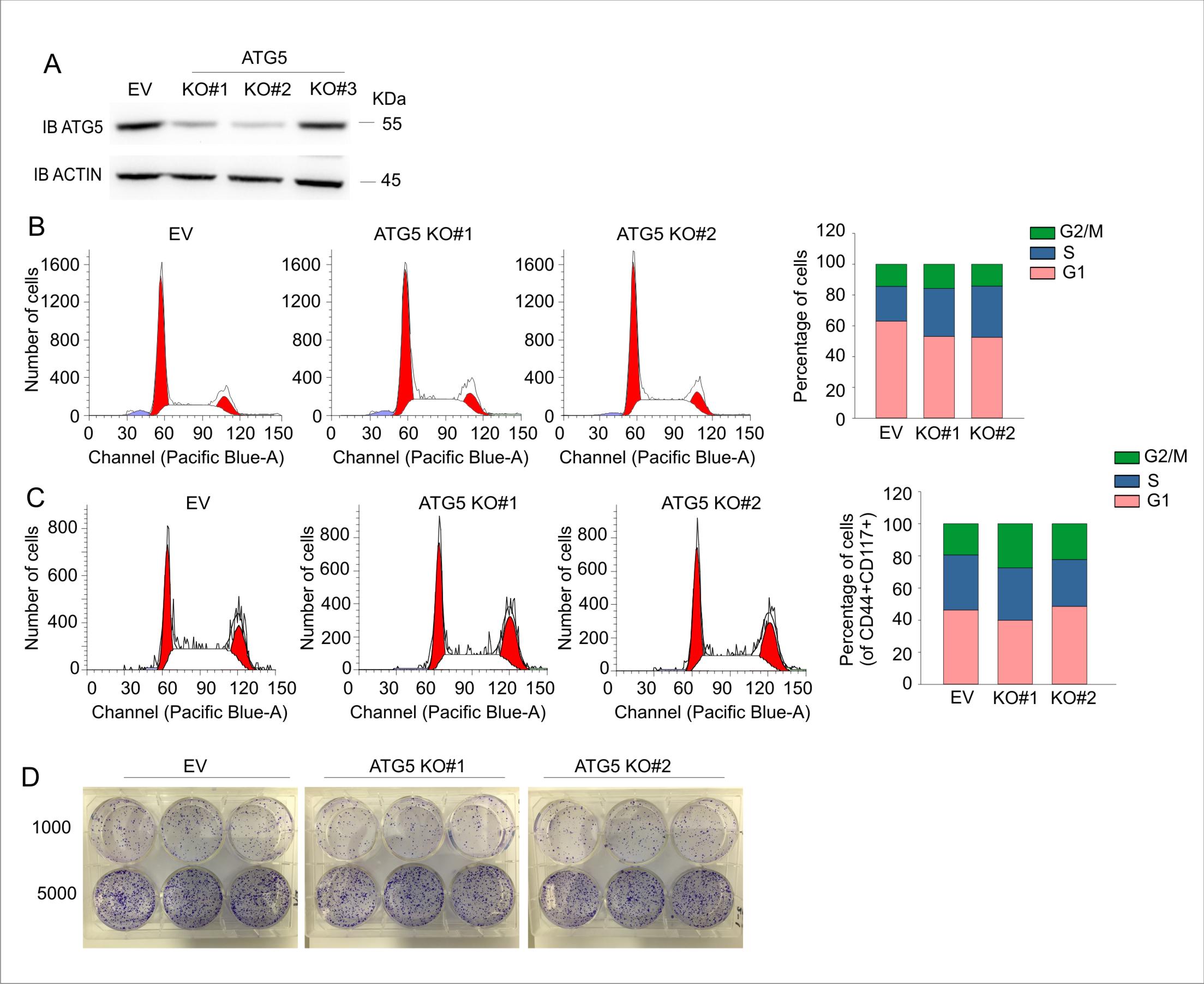


**Supplementary Figure S3. ATG5 knockout does not influence cell proliferation**

**A)** Western blot analysis of ATG5 expression in OVCAR-3 cells transduced with the empty vector (EV) or three ATG5-targeting vectors (KO#1, KO#2, and KO#3). Signal intensities of the bands were normalized against the actin signal. Only two (KO#1 and KO#2) out of three tested vectors significantly impaired ATG5 expression, and were chosen for subsequent experiments. One representative blot is shown.

**B)** Cell cycle analysis of OVCAR-3 cells transduced with EV, ATG5 KO#1, and KO#2 vectors. On the left, representative DNA content frequency histograms; on the right, graph showing the mean phase distribution of the cells (n=3 experiments).

**C)** Cell cycle analysis within the CSC compartment. On the left, representative DNA content frequency histograms; on the right, graph shows the mean phase distribution of the CD44^+^CD117^+^ cells (n=3 experiments).

**D)** Colony formation assay performed on EV, ATG5 KO#1-, and KO#2-transduced OVCAR-3 cells. Cells were plated either at the density of 1000 or of 5000 cells/well and stained with crystal violet after 10 days of culture. One representative picture is reported. No significant difference in the colony formation ability was observed.

**Supplementary Table**

| **Drug** | |  |  |
| --- | --- | --- | --- |
| **Carboplatin (μg/ml)** | **Chloroquine (μM)** | **Fractional Inhibition (*f*_a_)** | **CI** |
| (D)_1_ |  |  |  |
| 10 |  | 0.17 |  |
| 20 |  | 0.11 |  |
| 50 |  | 0.4 |  |
|  | (D)_2_ |  |  |
|  | 10 | 0.22 |  |
|  | 20 | 0.46 |  |
|  | 50 | 0.65 |  |
| (D)_1_+(D)_2_ |  |  |  |
| 10 | 10 | 0.26 | 1.17 |
| 10 | 20 | 0.44 | 0.67 |
| 10 | 50 | 0.58 | 0.52 |
| 20 | 10 | 0.45 | 0.98 |
| 20 | 20 | 0.52 | 0.85 |
| 20 | 50 | 0.72 | 0.45 |
| 50 | 10 | 0.66 | 1.05 |
| 50 | 20 | 0.69 | 0.99 |
| 50 | 50 | 0.8 | 0.62 |
|  |  |  |  |

**Supplementary Table S1.**

Combination index (CI) analysis of carboplatin and chloroquine treatment. Experimental data collected from n=3 EOC primary and PDX samples were subjected to automated calculation of CI using the software CompuSyn, as described in Materials and Methods. CI<1, C1=1, and CI>1 indicate synergism, additivity, and antagonism, respectively.
